# Ensemble-conditioned protein sequence design with Caliby

**DOI:** 10.1101/2025.09.30.679633

**Published:** 2025-10-20

**Authors:** Richard W. Shuai, Tianyu Lu, Subhang Bhatti, Petr Kouba, Po-Ssu Huang

**Affiliations:** Biophysics Program, Stanford University; Department of Bioengineering, Stanford University; Stanford Online High School; CIIRC, Czech Technical University in Prague, Loschmidt Laboratories, Masaryk University; Department of Bioengineering, Stanford University

## Abstract

Structure-conditioned sequence design models aim to design a protein sequence that will fold into a given target structure. Deep-learning-based approaches for sequence design have proven highly successful for various protein design applications, but many non-idealized backbones still remain out of reach for current models under typical *in silico* success criteria. We hypothesize that training objectives prioritizing native sequence recovery unintentionally push models to reproduce non-structural signals (e.g. phylogenetic relatedness, neutral drift, or dataset sampling biases), rather than a broadly generalizable structure-sequence mapping. Inspired by recent work bridging sequence likelihood and fitness prediction in protein language models, we introduce Caliby, a Potts model-based sequence design method capable of conditioning on an ensemble of structures. Conditioning on a synthetic ensemble generated from an input backbone allows sampling of sequences consistent with the structural constraints of the ensemble while averaging out undesired biases towards the native sequence. Ensemble-conditioned sequence design with Caliby reduces native sequence recovery while substantially improving AlphaFold2 self-consistency, outperforming state-of-the-art models ProteinMPNN and ChromaDesign on both native and *de novo* backbones. Finally, we train a variant of Caliby on only soluble proteins and demonstrate *in silico* that Protpardelle-1c binder designs that were previously deemed undesignable by SolubleMPNN are actually designable under SolubleCaliby, highlighting limitations of existing filtering pipelines. These results suggest that Caliby can expand the *de novo* design space beyond highly idealized backbones.

## 1 Introduction

*De novo* protein design aims to create proteins with desired functions by generating sequences that fold into specific structures [1]. Many design methods approach protein design by first sampling an initial backbone structure to capture an appropriate global fold, then designing sequences and sidechain conformations conditioned on these backbones [2]. Recent advances in deep generative modeling have made it possible to sample realistic protein backbones along with sequences that fold to them [3, 4].

A common *in silico* success metric for structure generative models is designability, which measures how closely designed sequences are predicted by a structure prediction model to fold back into the designed structures. However, recent analyses have shown that a large portion of native backbones fail this designability criterion, meaning that current models that optimize for designability are inherently unable to cover the full space of observed protein structures [5, 6]. As a result, many structure-generative methods currently struggle to produce proteins that are both diverse and robustly foldable outside a narrow, highly idealized subset of structures.

To address these challenges, we focus on improving structure-conditioned sequence design models to generate sequences that reliably fold to their target backbones, thereby expanding the space of designable backbones. We hypothesize that current structure-conditioned sequence design models learn to reproduce non-structural signals (e.g. phylogenetic relatedness, neutral drift, or dataset sampling biases) rather than a broadly generalizable structure-sequence mapping. In prior work, Ruffolo et al. show that as large structure-conditioned sequence design models are scaled, while their performance on native sequence recovery benchmarks continuously improves, their ability to produce sequences that are predicted to fold back into the target structure degrades, approaching the low success rates of native sequences [7]. We posit that this tradeoff arises because models learn to capture non-structural, evolutionary signal from the training data, which dilutes the specific structural signal required for a sequence to robustly fold into the target backbone.

Similar trends have been observed in scaling protein language models (pLMs) for fitness prediction, with larger pLMs achieving lower perplexity scores but plateaued or lowered variant effect prediction accuracies [8–10]. Pugh *et al*. address this gap between sequence likelihood and fitness prediction by introducing a simple strategy, Likelihood-Fitness Bridging (LFB), which averages model scores across sequences subject to similar selective pressures to average out phylogenetic noise, finding that this strategy improves variant effect prediction accuracies for large pLMs [11].

Drawing inspiration from LFB, we introduce Caliby, a Potts model-based sequence design method capable of conditioning on an ensemble of structures. Given an input backbone, we generate a synthetic ensemble with partial diffusion using a backbone generative model, allowing us to sample sequences consistent with the structural constraints of the synthetic ensemble while averaging out undesired biases towards native sequence recovery. We demonstrate that this strategy allows us to obtain sequences that more precisely match native backbone structures, as predicted by single-sequence AlphaFold2 [12], while simultaneously reducing native sequence recovery. Furthermore, these results generalize to better *in silico* designability on *de novo* backbones and reveal viable binder designs from Protpardelle-1c previously overlooked by SolubleMPNN.

Our results show that ensemble-conditioned Caliby successfully designs sequences compatible with all structural constraints within a given synthetic ensemble. Importantly, the principles underlying our ensemble-conditioning approach are not limited to synthetic ensembles and can be extended to any user-provided ensemble. Therefore, beyond improving designability, we anticipate that Caliby can be used for robust multistate sequence design across a wider range of structural constraints defined either by experimental or computational ensembles. To enable broader community access, we plan to make model weights, sampling code, and training code for Caliby available at https://github.com/ProteinDesignLab/caliby.

## 2 Related Work

The goal of structure-conditioned sequence design is to design a sequence that will reliably fold back into the given target structure [4]. Classical approaches to this problem include modeling an energy landscape and performing combinatorial optimization over sequence identity and sidechain rotamers to identify low-energy configurations [13, 14]. Early methods, such as Rosetta backrub, have been used to generate structural ensembles from crystal structures to allow flexible backbone sequence design [15–17].

Several deep-learning-based methods have emerged as a powerful alternative to classical approaches [4, 7, 18–32]. These methods often use a graph neural network (GNN) to encode an input backbone structure, followed by autoregressive sequence decoding or iterative sequence unmasking [4, 7, 18, 21, 24, 28, 31]. Other approaches sample from an energy landscape defined by a neural-network-derived Potts model [25, 29, 30] or jointly optimize sequence design and structure prediction to achieve inverse folding [33]. Among all of these methods, ProteinMPNN remains the dominant model for sequence design due to its demonstrated robust performance on real-world design tasks [4, 34–36]. Related to our work, Akiyama and Ovchinnikov investigated ProteinMPNN sequence design on individual structures of an ensemble generated by RFdiffusion, showing that these sequences exhibit increased diversity without compromising AlphaFold2 designability [37].

Deep-learning-based approaches have also been extended to condition on multiple input structures. Praetorius *et al*. and Guo *et al*. average logits predicted by ProteinMPNN for two separate states to design two-state proteins [38, 39]. ProteinGenerator generates sequences with diffusion and can incorporate multiple structural constraints [40]. Most recently, DynamicMPNN explicitly conditions on multiple input structures from a conformational ensemble to perform sequence design [41].

## 3 Methods

### 3.1 Model architecture

**Figure 1:**
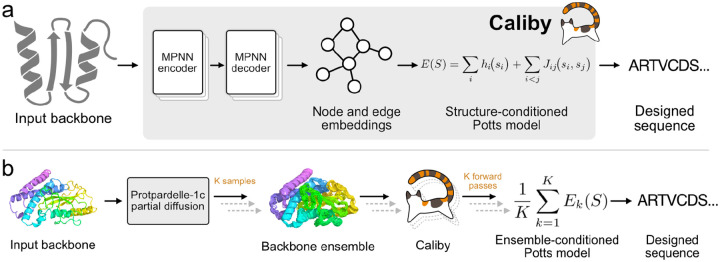
(**a**) Caliby designs sequences onto an input backbone by sampling from a structure-conditioned Potts model derived from a neural network. (**b**) Caliby can condition on an ensemble of structures produced by a backbone generative model by averaging the sitewise and pairwise energy terms of the Potts models derived from each input structure.

Our model architecture builds on the ProteinMPNN architecture, consisting of 3 MPNN encoder layers followed by 3 MPNN decoder layers (Figure 1a) [4]. Similar to Full-Atom MPNN (FAMPNN), we remove the causal mask from the MPNN decoder layers and expand the featurization to encode pairwise distances between sidechain atoms if sidechains are provided as context [28].

### 3.2 Sequence prediction

For sequence prediction, we adapt the ChromaDesign Potts decoder module, which projects node and edge embeddings from a graph neural network to predict the sitewise and pairwise energy terms of a Potts model [25]. With a single pass, Caliby produces a Potts model for a single structure, where the probability of a sequence can be expressed as:

$$p(S)=\frac{1}{Z}\text{exp}(-E(S)),\;E(S)=\sum_{i}h_{i}\left(s_{i}\right)+\sum_{i<j}J_{ij}\left(s_{i},s_{j}\right),\;Z=\sum_{S}\text{exp}(-E(S))$$

Here, $h_{i}$ are sitewise terms (fields) that score individual residue identities at site i and $J_{ij}$ are pairwise energy terms (couplings) that capture interaction preferences between residue pairs at sites i and j. We sample sequences from this neural-network-derived Potts model using the Discrete Langevin Monte Carlo (DLMC) algorithm with an additional local composition perplexity (LCP) restraint to penalize low complexity sequences, as done in ChromaDesign [25, 42].

### 3.3 Ensemble design

We express the energy of a sequence conditioned on a structural ensemble of K structures as the average energy of the sequence scored by each of the Potts models derived from each input structure (Figure 1b):

$$E_{\text{ens}}(S)=\frac{1}{K}\sum_{k=1}^{K}E_{k}(S)$$

This is equivalent to sampling from a single Potts model with sitewise and pairwise energy terms averaged over the Potts models computed for each structure in the ensemble. As a result, once the averaged Potts model is precomputed for a given ensemble, sequences can be sampled for the ensemble at the same speed as sampling for a single structure (Appendix B.2).

With this formulation, the ensemble-conditioned sequence distribution is the normalized geometric mean of the per-structure sequence distributions (i.e. $p_{\text{ens}}(S)\propto \prod_{k}p_{k}(S{)}^{1/K}$), which is closely related to a Product-of-Experts (PoE) model (Appendix B.2.2) [43]. We can therefore interpret ensemble design as sampling from the distribution of sequences that are compatible with all structures in the ensemble.

To generate synthetic ensembles, we use Protpardelle-1c partial diffusion with 150 rewind steps out of 500 total steps to generate 15 additional backbone conformers (K=16), although we expect our method to be general to other methods for generating backbone ensembles, such as Rosetta backrub or RFdiffusion [3, 16, 44]. We find that K=16 provides good results, with slightly improved but diminishing returns for larger ensemble sizes (Appendix Figure A5).

## 4 Results

### 4.1 Ensemble design improves the structural signal present in designed sequences

**Figure 2:**
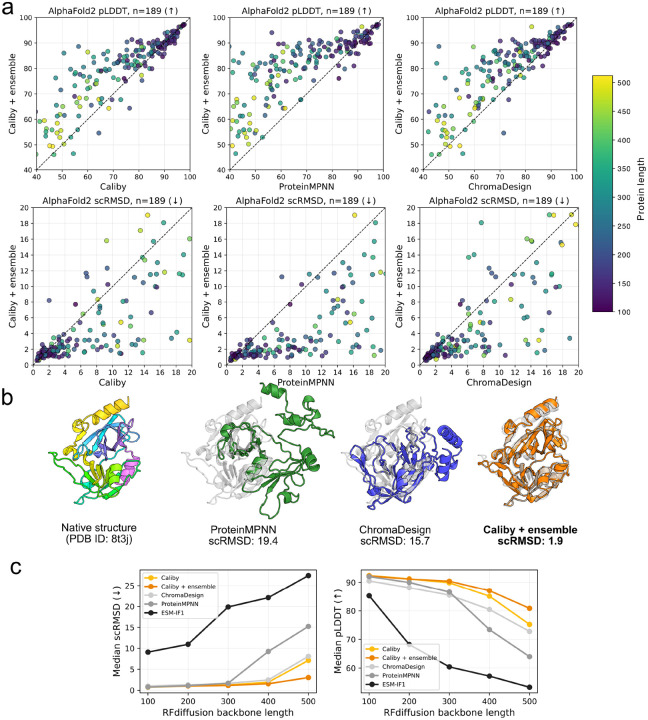
(**a**) Evaluation of single-sequence AlphaFold2 self-consistency on a test set of 85 native backbones from lengths 100 to 1000. The best of 8 designed sequences is plotted for each backbone. Full plots annotated with PDB IDs are shown in Appendix Figure A1. (**b**) Visualized example of a native backbone that ProteinMPNN (green) and ChromaDesign (blue) fail to design but that ensemble-conditioned Caliby (orange) can successfully design. (**c**) Median self-consistency RMSDs (left) and pLDDTs (right) for sequence design methods across 100 RFdiffusion backbones for each length in {100, 200, 300, 400, 500}.

We evaluated the structural signal present in sequences designed by each model by computing self-consistency RMSD (scRMSD) and pLDDT with single-sequence AlphaFold2. To evaluate performance on native backbones, we used protein monomers between lengths 100 and 1024 from the Boltz-1 test set [45]. We designed 8 sequences for each backbone using Caliby, ProteinMPNN, ChromaDesign, and ensemble-conditioned Caliby, taking the best of 8 sequences. We found that ensemble-conditioned Caliby achieves better self-consistency on native backbones compared with all other methods (Figure 2a) and that challenging native backbones with previously low designability can be successfully designed by ensemble-conditioned Caliby (Figure 2b).

To evaluate on *de novo* backbones, following the benchmark established in ProteinBench, we generated 100 RFdiffusion-generated backbones for each length in {100, 200, 300, 400, 500} and computed self-consistency metrics for each model, finding that ensemble-conditioned Caliby is often able to find sequences that are predicted to fold back into the designed structures even at longer RFdiffusion lengths of 400 and 500 (Figure 2c) [46].

**Figure A2:**
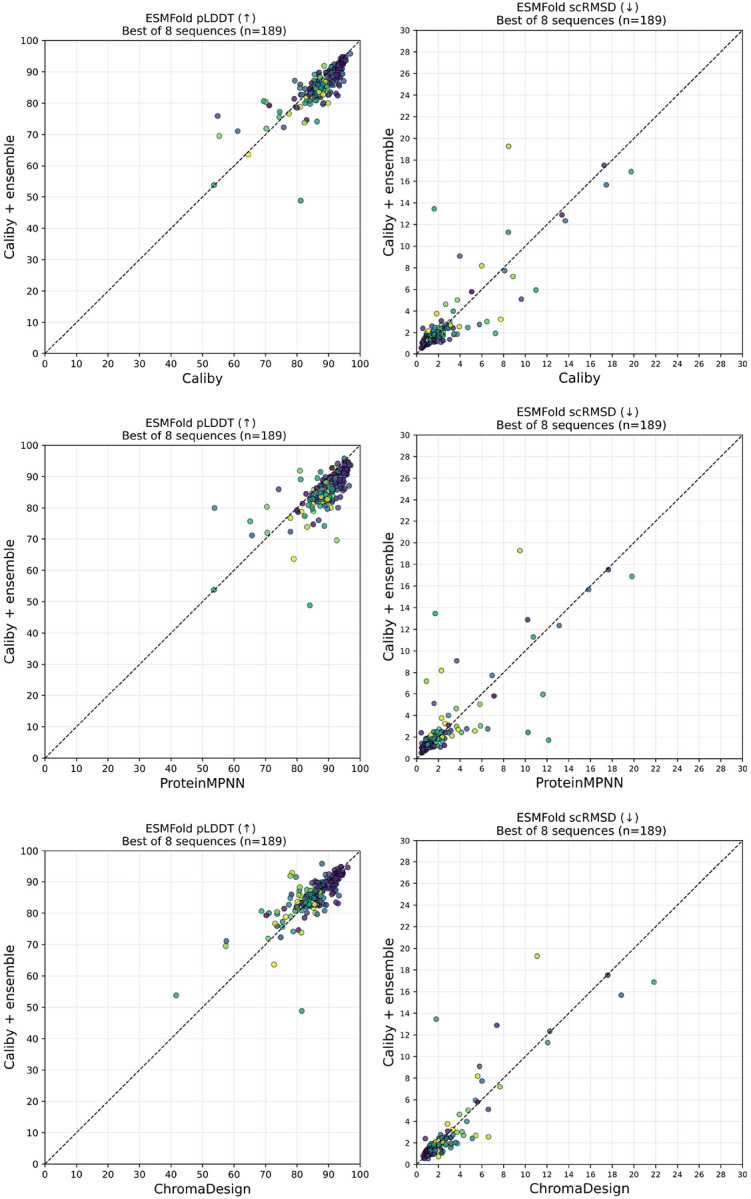
ESMFold self-consistency metrics on a test set of 85 native backbones, annotated with the PDB ID of each structure.

**Figure A3:**
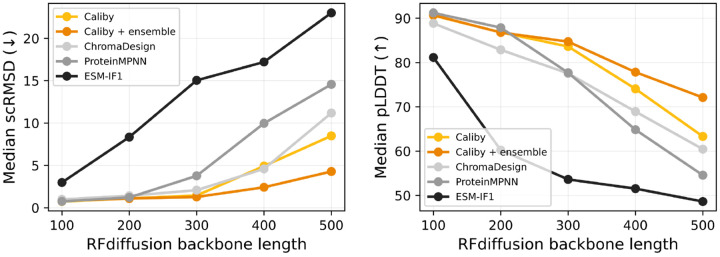
Median ESMFold self-consistency metrics across 100 RFdiffusion backbones for each length in {100, 200, 300, 400, 500}.

**Figure A5:**
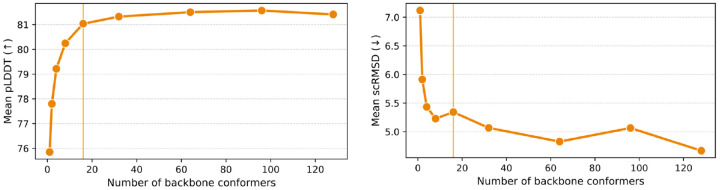
Mean pLDDT (left) and mean scRMSD (right) of the best of 8 sequences across a test set of 85 native backbones as a function of the number of backbone conformers provided to Caliby. The orange vertical line represents 16 backbone conformers (K=16).

We also evaluated self-consistency metrics using ESMFold (Appendix Figures A2,A3) [47]. We found that while ESMFold is often able to correctly predict the native structure regardless of the sequence design method, it struggles slightly more than single-sequence AlphaFold2 to fold sequences designed on RFdiffusion *de novo* backbones. On the *de novo* backbones, we found a similar trend that ensemble-conditioned Caliby performs best, but on native backbones, all models perform similarly, potentially due to less room for improvement.

### 4.2 Ensemble design reduces bias towards the native sequence

**Figure 3:**
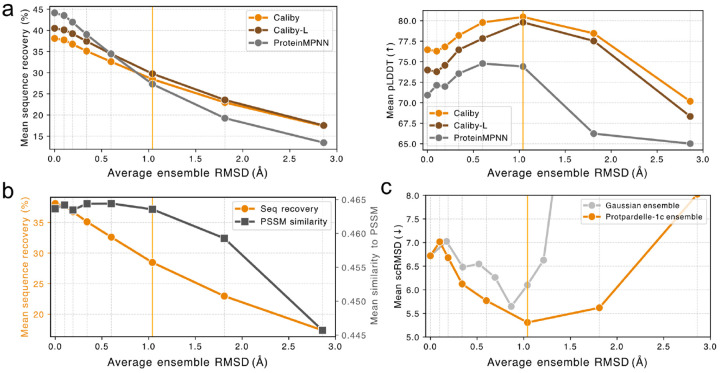
(**a**) Mean sequence recovery and mean AlphaFold2 pLDDT (best of 8) vs. ensemble structural diversity, for Caliby, Caliby-L, and ProteinMPNN. (**b**) Mean sequence recovery (orange) and mean PSSM similarity (dark gray, measured as 1 - Jensen-Shannon divergence of the PSSM to the designed sequence profile) vs. ensemble structural diversity. (**c**) Mean scRMSD (best of 8) over native backbones vs. ensemble structural diversity, using either Protpardelle-1c (orange) or Gaussian noise (light gray) ensemble generation. Vertical grid lines represent the number of rewind steps for Protpardelle-1c partial diffusion: [0, 50, 75, 100, 125, 150, 175, 200], with the orange line representing 150 rewind steps.

To examine the sequence properties of ensemble-conditioned design, we used Caliby to design sequences onto the native backbones either with no ensemble (Figure 3, 0Å) or with Protpardelle-1c ensemble generation with different numbers of partial diffusion rewind steps. Increasing rewind steps results in increased structural variation within the ensemble, which we quantify for analysis by computing the average RMSD of the resulting partial diffusion structures to the starting structure.

We found that ensemble-conditioned design considerably reduces native sequence recovery compared with no ensemble design while improving pLDDT of the designed sequences (Figure 3a). With too many rewind steps (175 or more), we see a reduction in pLDDT of predicted sequences, indicating that the structural ensemble becomes too diverse to be beneficial for sequence design.

We also trained a larger version of Caliby, Caliby-L, where we increased the number of MPNN layers from 3 to 5 and increased the hidden dimension from 128 to 256. We found that while Caliby-L achieves better test set sequence recovery than Caliby, it designs sequences with worse pLDDT, suggesting that increased model capacity does not necessarily contribute to learning a stronger structural signal. However, with ensemble-conditioning, we can improve the pLDDT of designs from Caliby-L to levels approaching that of Caliby while similarly reducing native sequence recovery, providing evidence that ensemble-conditioned design can average out non-structural signal learned by larger models (Figure 3a).

We also performed ProteinMPNN tied sampling across synthetic ensembles and found a similar improvement in self-consistency accompanied by reduced native sequence recovery, further supporting our hypothesis that ensemble-conditioned design can more generally be used to average out non-structural signal (Figure 3a). Tied sampling is similar to ensemble-conditioned Caliby in that for a given decoding step, averaging logits across multiple structures samples from the normalized geometric mean of the next-token distributions predicted from each structure (Appendix B.2.3). However, Caliby performs better overall and scales more efficiently with K: for a protein of length N, each additional conformer in ProteinMPNN adds N forward passes per sampled sequence, whereas Caliby needs only one extra forward pass, after which sampling cost is independent of K.

**Figure A6:**
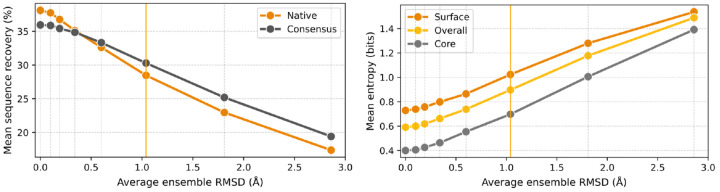
Native and consensus sequence recovery (left) and sequence entropy (right) as a function of average ensemble RMSD. Vertical grid lines represent the number of rewind steps for Protpardelle-1c partial diffusion: [0, 50, 75, 100, 125, 150, 175, 200], with the orange line representing 150 rewind steps.

In Figure 3b, we show that while native sequence recovery drops with increasing average ensemble RMSD, the similarity of the designed sequence profile to the position-specific scoring matrix (PSSM) of the multiple sequence alignment (MSA) initially remains relatively constant, showing that although designed sequences capture less of the native sequence, they still reflect statistics of the MSA. We also found that the diversity of designed sequences increases with ensemble design, indicating that ensemble design is not simply converging on a single highly confident sequence and that diversity and designability can simultaneously be improved with ensemble-conditioned design (Appendix Figure A6).

Finally, in Figure 3c, we show that Gaussian noise can potentially also be used as a cheap method for ensemble generation, although less structural variation is tolerated than when using Protpardelle-generated ensembles, which are more physically realistic.

### 4.3 SolubleCaliby identifies designable binders missed by SolubleMPNN

Similar to SolubleMPNN, we also trained SolubleCaliby, a variant of Caliby trained by excluding transmembrane proteins from the training set (Appendix A) [48]. To illustrate its effectiveness, we revisited binder designs originally described and generated by Protpardelle-1c for various BindCraft targets [44]. These binders were initially produced by target-conditioned backbone generation with Protpardelle-1c, followed by AlphaFold2-Multimer hallucination for designing interface residues and SolubleMPNN for designing non-interface residues. Similar to BindCraft, these designs were then evaluated using single-sequence AlphaFold2 to predict designability metrics for both the complex and the binder alone. We hypothesized that many inherently viable Protpardelle-1c designs might have been incorrectly dismissed as undesignable when evaluating using SolubleMPNN, and that SolubleCaliby could recover a wider set of viable binder designs.

**Figure 4:**
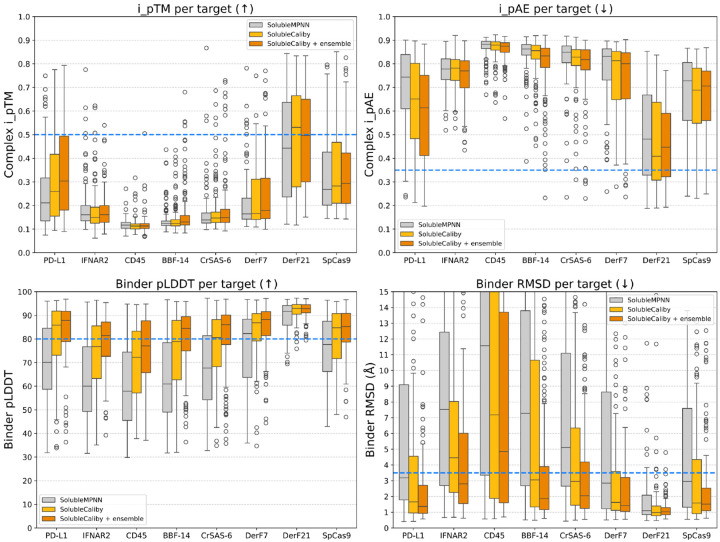
SolubleMPNN vs. SolubleCaliby vs. ensemble-conditioned SolubleCaliby used for designing non-interface residues on binders designed by Protpardelle-1c for various BindCraft targets. Success thresholds are denoted as blue dashed lines (i_pTM > 0.5, i_pAE < 0.35, binder pLDDT > 0.8, binder RMSD < 3.5 Å).

**Figure 5:**
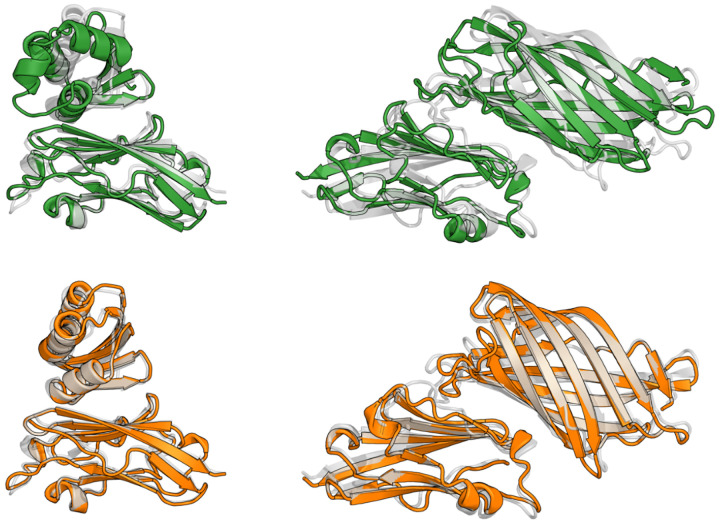
Examples of Protpardelle-1c designs (gray) targeting PD-L1 that failed with SolubleMPNN (green) but succeeded with ensemble-conditioned SolubleCaliby (orange).

**Figure A7:**
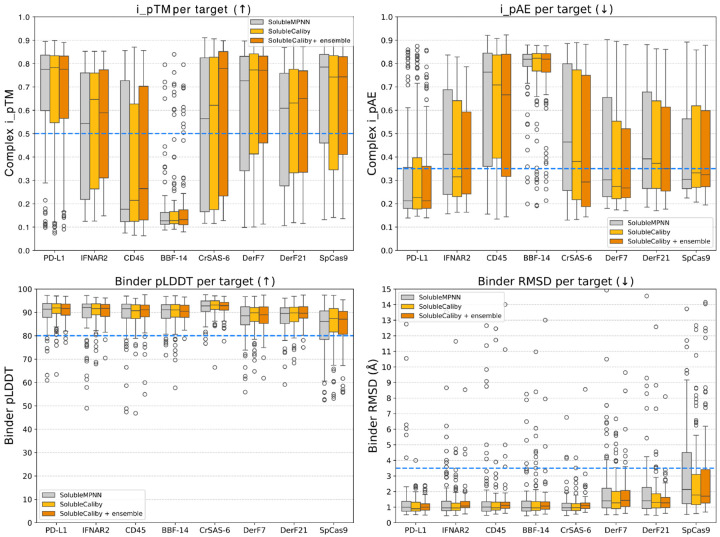
SolubleMPNN vs. SolubleCaliby vs. ensemble-conditioned SolubleCaliby used for designing non-interface residues on binders from raw BindCraft trajectories. Success thresholds are denoted as blue dashed lines (i_pTM > 0.5, i_pAE < 0.35, binder pLDDT > 0.8, binder RMSD < 3.5 Å)

To explore this, we redesigned the non-interface residues for this set of binders, observing that using SolubleCaliby instead of SolubleMPNN markedly improves designability metrics (Figure 4), especially for the binder-only self-consistency metrics (Binder pLDDT and Binder RMSD). Figure 5 shows two examples of Protpardelle-1c designs that failed under SolubleMPNN but succeeded with ensemble-conditioned SolubleCaliby. By contrast, when redesigning binders from BindCraft trajectories, SolubleMPNN and SolubleCaliby perform similarly, likely because hallucinated binders designed by BindCraft trajectories tend to be highly idealized and are already designable with SolubleMPNN (Appendix Figure A7). Overall, these results suggest that Caliby can expand the possible protein design space, successfully designing not only highly idealized binders but also those previously considered undesignable by ProteinMPNN.

## 5 Discussion

Designability metrics based on single-sequence AlphaFold2 prediction have become popular for filtering designs intended for experimental characterization. In this work, we introduced Caliby, a structure-conditioned sequence design method that considerably improves *in silico* success rates.

The ability of Caliby to effectively handle structural ensembles is promising for applications in multistate design, where Caliby may be used to design sequences to simultaneously accommodate multiple structural conformations. However, in these cases, single-sequence AlphaFold2 alone may be an ineffective filter for successful designs. While AlphaFold2 is valuable for guiding model development, strict reliance on AlphaFold2-based metrics might exclude sequences that can successfully fold or function experimentally. Future work can both explore multistate design with Caliby and investigate whether ensemble-conditioned Caliby learns an energy function distinct from that learned by AlphaFold2. These investigations may reveal new metrics for predicting experimental success based on Caliby, enabling designs beyond those identifiable by AlphaFold2 alone.

Beyond the immediate practical applications of Caliby, we used single-sequence AlphaFold2 designability as a proxy to assess how easily the structural signal can be extracted from a designed sequence. Our analysis demonstrates that ensemble-conditioned design can disentangle true structural signal from confounding non-structural signal, such as phylogenetic relationships, sampling biases, or various selective pressures not identifiable from backbone structure alone. Additionally, similar to the rationale described by Dauparas *et al*. for adding small amounts of Gaussian noise during ProteinMPNN training, ensemble-conditioned Caliby may implicitly average out experimental artifacts, ensuring that input ensembles better reflect physically relevant conformations encountered outside crystallographic conditions [4]. Overall, our findings challenge a common practice of optimizing for native sequence recovery as the main objective in structure-conditioned sequence design models and suggest that sequence design models may face similar scaling behaviors as recently observed with protein language models. We hope that our work can inform future research on understanding the information learned by structure-conditioned sequence design models.

## Figures and Tables

**Figure A1:**
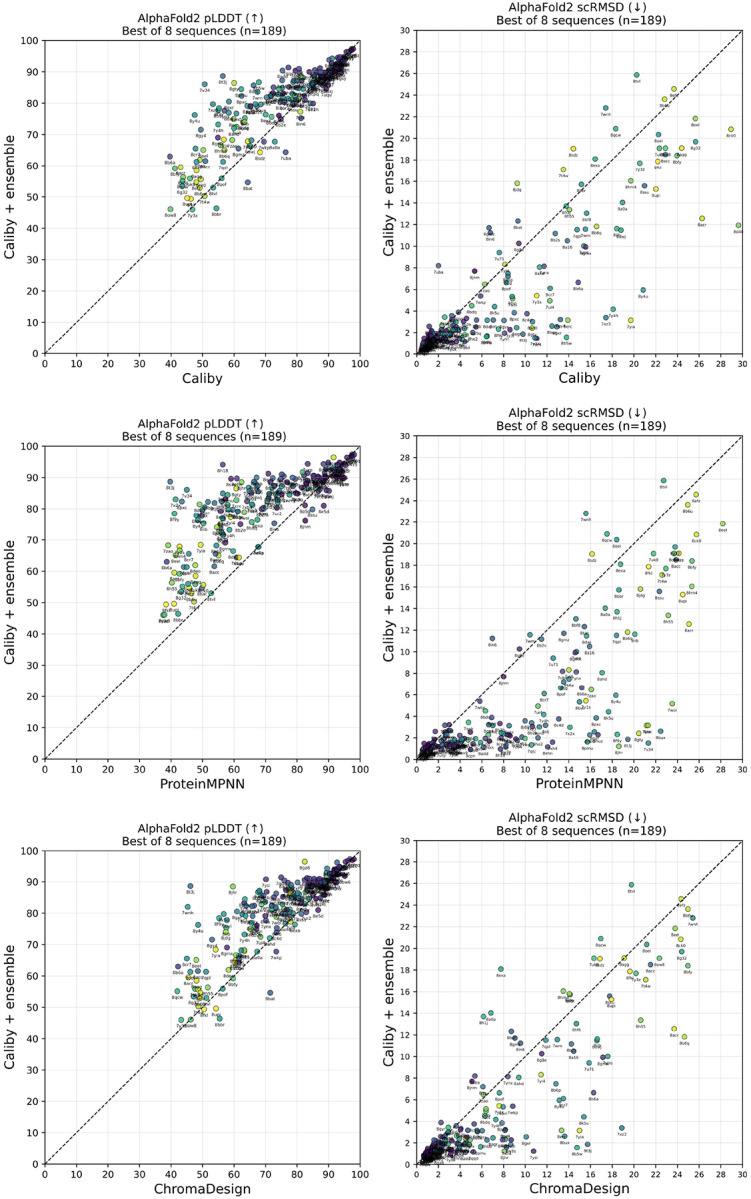
Full versions of plots from Figure 2a. AlphaFold2 self-consistency metrics on a test set of 85 native backbones.

**Figure A4:**
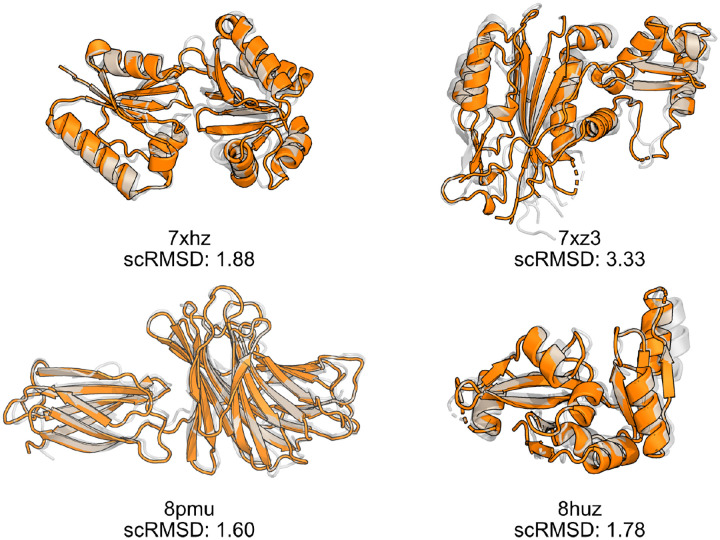
Additional examples of challenging native backbones (gray) and the AlphaFold2 predictions using the best of 8 sequences designed by ensemble-conditioned Caliby (orange).

## A Datasets

Our dataset curation closely follows the Boltz-1 procedure [45]. For our training set, we used all PDB structures released before 2021-09-30 with a resolution less than 9Å [49]. We developed methodology using the validation set curated by Boltz-1, which uses MMseqs2 to cluster sequences at a 40% sequence identity threshold to ensure that no sequences in the validation set fall into any train clusters [50]. Then, the validation set is filtered for structures with resolution below 4.5Å and released between 2021-09-30 and 2023-01-13. The test set is constructed similarly to the validation set, but only using structures released after 2023-01-13.

For data preprocessing and data loading, we adopted the AtomWorks framework to parse the first bioassembly of each PDB and clean RCSB structures [51]. During training, we sampled protein chains weighted inversely proportionally to the size of the cluster that the chain falls into, and applied random contiguous cropping to the chain if the chain is longer than 1024 residues. We found that despite being trained on single chains, Caliby generalizes well to sequence design on protein complexes. From our preliminary experiments, we did not find evidence that training on multiple chains measurably improved results despite testing various modes of spatial cropping along interfaces, so we chose to simplify training and leave explicit interface training for future work.

For training SolubleCaliby, we downloaded all transmembrane proteins excluded from SolubleMPNN training provided on the ProteinMPNN GitHub at https://github.com/dauparas/ProteinMPNN/blob/main/soluble_model_weights/excluded_PDBs.csv [48]. We additionally excluded any transmembrane PDB codes annotated in the Protein Data Bank of Transmembrane Proteins at https://pdbtm.unitmp.org/downloads [52].

## B Model details

### B.1 Training details

Caliby was trained for 30,000 steps with 4 gradient accumulation steps each with a batch size of 8, taking about 20 hours on a single NVIDIA H100 GPU with 80GB of RAM. We kept an exponential moving average (EMA) of model parameters with a decay of 0.99 to use for evaluation [53].

Similar to ChromaDesign and TERMinator, we trained Caliby to condition on an input structure and predict a set of Potts parameters over the sequence that optimizes the composite pseudo-likelihood of the native sequence [25, 29, 54]. During training, we added Gaussian noise with standard deviation 0.3Å to the input structure coordinates [4]. Following FAMPNN, we also provided partial sequence and sidechain context to the model during training so that users can condition on mixtures of sequence-only and sequence-and-sidechain context. Specifically, we sampled $p_{1},p_{2}\sim \text{Uniform}(0,1)$ for each training example and unmasked each residue identity in the native sequence with probability $p_{1}$. For each residue whose identity was unmasked, we unmasked the native sidechain conformation with probability $p_{2}$.

### B.2 Ensemble design details

Here, we provide additional details regarding the ensemble Potts model formulation used by Caliby for structural-ensemble-conditioned sequence design. Given an ensemble of K structures, we first compute the neural-network-derived Potts model independently for each structure indexed by k:

$$p_{k}(S)=\frac{1}{Z_{k}}\text{exp}\left(-E_{k}(S)\right),\;E_{k}(S)=\sum_{i}h_{i}^{\left(k\right)}\left(s_{i}\right)+\sum_{i<j}J_{ij}^{\left(k\right)}\left(s_{i},s_{j}\right)$$

where $Z_{k}$ is the normalizing factor known as the partition function. In practice, we also tranform each Potts model to zero-sum gauge so that:

(1)
$$\sum_{a\in 𝒜}h_{i}^{\left(k\right)}(a)=0,\;\sum_{a\in 𝒜}J_{ij}^{\left(k\right)}(a,b)=0,\;\sum_{b\in 𝒜}J_{ij}^{\left(k\right)}(a,b)=0$$

where 𝒜 is the alphabet of amino acids. Then, given K input structures with Potts energies $E_{k}$, we define the ensemble energy as the average:

$$E_{\text{ens}}(S)=\frac{1}{K}\sum_{k=1}^{K}E_{k}(S)$$

When designing on ensembles of K structures, we include the original structure as the first backbone and use partial diffusion structures for the remaining K-1 structures.

#### B.2.1 Ensemble design as an average of Potts models

By linearity, $E_{\text{ens}}$ is itself a Potts energy computed from Potts parameters equal to the average of the per-structure parameters:

(2)
$$E_{\text{ens}}(S)=\frac{1}{K}\sum_{k=1}^{K}E_{k}(S)$$

(3)
$$=\frac{1}{K}\sum_{k=1}^{K}\left(\sum_{i}h_{i}^{\left(k\right)}\left(s_{i}\right)+\sum_{i<j}J_{ij}^{\left(k\right)}\left(s_{i},s_{j}\right)\right)$$

(4)
$$=\frac{1}{K}\left(\sum_{i}\sum_{k=1}^{K}h_{i}^{\left(k\right)}\left(s_{i}\right)+\sum_{i<j}\sum_{k=1}^{K}J_{ij}^{\left(k\right)}\left(s_{i},s_{j}\right)\right)\;(\text{swap sums})$$

(5)
$$=\sum_{i}\left(\frac{1}{K}\sum_{k=1}^{K}h_{i}^{\left(k\right)}\right)\left(s_{i}\right)+\sum_{i<j}\left(\frac{1}{K}\sum_{k=1}^{K}J_{ij}^{\left(k\right)}\right)\left(s_{i},s_{j}\right)$$

As a result, by simply averaging the Potts parameters across the input structures, we can repeatedly sample sequences for the ensemble at the same rate as sampling for a single structure.

Since precomputing the Potts parameters for each structure requires only a single pass through the neural network, the sampling time is dominated by sequence sampling from the Potts model. In practice, on a backbone of length 500, computing Potts parameters takes roughly 0.05 seconds per input structure and 0.8 seconds to sample each sequence from the averaged Potts model on an NVIDIA H100.

#### B.2.2 Ensemble-conditioned sampling as a normalized geometric mean

Ensemble-conditioned Caliby can be seen as sampling sequences from a normalized geometric mean over the per-structure sequence distribitions. If the ensemble energy is the average

$$E_{\text{ens}}(S)=\frac{1}{K}\sum_{k=1}^{K}E_{k}(S),$$

then

$$p_{\text{ens}}(S)\propto \text{exp}\left(-\frac{1}{K}\sum_{k=1}^{K}E_{k}(S)\right)={\left(\prod_{k=1}^{K}e^{-E_{k}(S)}\right)}^{1/K}\propto \prod_{k=1}^{K}p_{k}(S{)}^{1/K}$$

so the ensemble Potts distribution is proportional to the geometric mean of the per-structure Potts distributions.

#### B.2.3 ProteinMPNN tied sampling as a per-step normalized geometric mean

At each decoding step, ProteinMPNN tied sampling over an ensemble is a normalized geometric mean of the per-structure distributions for the next token. At decoding step t, for a given amino acid a, structure k yields logits $z_{a}^{\left(k\right)}$ with

$$p^{\left(k\right)}(a)=\frac{e^{z_{a}^{\left(k\right)}}}{Z^{\left(k\right)}},\;Z^{\left(k\right)}=\sum_{b}e^{z_{b}^{\left(k\right)}}.$$

The tied distribution at decoding step t is obtained by averaging logits, then applying softmax:

$$p_{\text{ens}}(a)=\text{softmax}\left(\frac{1}{K}\sum_{k}z_{a}^{\left(k\right)}\right)=\frac{e^{\frac{1}{K}\sum_{k}z_{a}^{\left(k\right)}}}{\sum_{b}e^{\frac{1}{K}\sum_{k}z_{b}^{\left(k\right)}}}=\frac{{\left(\prod_{k}e^{z_{a}^{\left(k\right)}}\right)}^{1/K}}{\sum_{b}{\left(\prod_{k}e^{z_{b}^{\left(k\right)}}\right)}^{1/K}}$$

From the previous line, the denominator is independent of the choice of amino acid a and can be treated as a normalization constant, so we can see that

$$p_{\text{ens}}(a)\propto {\left(\prod_{k}p^{\left(k\right)}(a)\right)}^{1/K}$$

meaning that at each decoding step, the tied sampling distribution is a normalized geometric mean over the next-token distributions predicted from each structure. We note that unlike with ensemble-conditioned Caliby, this property does not necessarily hold for the whole sequence distribution.

In practice, for ProteinMPNN tied sampling, we used the implementation provided by the Kuhlman Lab at https://github.com/Kuhlman-Lab/proteinmpnn.

## C Evaluation

### C.1 Self-consistency evaluation

For all AlphaFold2 evaluations, we use AlphaFold2 in single-sequence mode with 3 recycles, taking the best by pLDDT out of 5 models, as implemented in ColabDesign [55]. We ran ProteinMPNN sequence design with the 0.2Å checkpoint (vanilla_model_weights/v_48_020.pt) and all other sampling parameters at their defaults. We ran ChromaDesign using the publicly available weights and all sampling parameters at their defaults (t=0.5).

To select native backbones for self-consistency evaluation, we used the first bioassembly for each structure in the Boltz-1 test set and extracted all protein monomers between lengths 100 and 512, yielding 194 protein chains. We excluded 5 PDB codes due to issues with consistent parsing between different sequence design methods, resulting in 189 chains for evaluation.

### C.2 Designed sequence profile analysis

We sampled 128 sequences per PDB with Caliby to analyze the sequence recovery and designed sequence profiles. To compare against MSA statistics, we generated MSAs using ColabFold [56], which uses MMseqs2 for sequence search.

### C.3 Bindcraft targets benchmark

For evaluating Protpardelle-1c designs, we used the publicly available samples generated for the BindCraft benchmark at https://zenodo.org/records/17096818. For evaluating on BindCraft designs, we took a random subsample of 100 passing BindCraft trajectories per target and re-designed non-interface residues using either SolubleCaliby or SolubleMPNN with the same settings as in BindCraft. Following Lu *et al*., we predicted designs using single-sequence AlphaFold2 and used the template of the target, removed template of the binder, removed interchain template features, did not use initial guess, and removed target template sequence features to allow for target backbone flexibility [44].
